# Analysis of influencing factors of traffic accidents on urban ring road based on the SVM model optimized by Bayesian method

**DOI:** 10.1371/journal.pone.0310044

**Published:** 2024-09-24

**Authors:** Lei Wang, Mei Xiao, Jiliang Lv, Jian Liu

**Affiliations:** 1 School of Automobile, Chang’an University, Xi’an, China; 2 School of Automobile & Rail Transportation, Tianjin Sino-German University of Applied Sciences, Tianjin, China; 3 College of Transportation Engineering, Chang’an University, Xi’an, China; 4 Automotive Data of China Co. Ltd., China Automotive Technology and Research Center Co., Ltd., Tianjin, China; Vietnam Maritime University, VIET NAM

## Abstract

Based on small scale sample of accident data from specific scenarios, fully exploring the potential influencing factors of the severity of traffic accidents has become a key and effective research method. In order to analyze the factors mentioned above in the scenario of urban ring roads, this paper collected data records of 1250 traffic accidents involving different severity on urban ring road of a central city in northwest China in the past 3 years. Firstly, the Support Vector Machine (SVM) model of non-parametric method is utilized to analyze the data above, and three kernel functions of linear, inhomogeneous polynomial and Gaussian radial basis are constructed respectively. Considering comprehensively 16 potential influencing factors covering the driver-vehicle-road-environment integrated system, the SVM models of above three kernel functions are verified, accuracy reaches 0.771 and F1 reaches 0.841. Then, Bayesian Optimization (BO), Grids Search (GS) and Rough Set (RS) are utilized as optimizer to adjust the parameters of Gaussian radial basis function SVM model, the performance of BO-SVM is further improved and reaches the optimum, with an average accuracy of 0.875 on the test set and a F1 of 0.886, completely outperforming the benchmark models of GS-SVM, RS-SVM, Bilayer-LSTM and BP. Finally, the sensitivity analysis method is utilized to quantify the sensitivity of the potential influencing factors to the severity of road accidents, and the backward selection method is utilized to screen the core influencing factors that influence the severity of accident, concluded that core influencing factors are age, driving mileage and vehicle type. This paper will provide reference for the analysis of the significant influencing factors for road accidents severity, and to provide theoretical support for the precise formulation of accident improvement strategies.

## 1. Introduction

The study of road traffic accidents serves as a cornerstone for the development of effective traffic system safety countermeasures. The complexity of the causes underlying road traffic accidents necessitates a thorough examination of the factors that influence their severity. Drawing insights from accident data to uncover the roots of traffic accident severity has emerged as a promising research theory and method.

Analyzing the intricate causes of traffic accidents necessitates a thorough examination of the pivotal factors that govern the severity of road incidents. This comprehensive understanding allows for the precise formulation of targeted traffic safety enhancement strategies, thereby effectively reducing the detrimental consequences of accidents and safeguarding the personal well-being and property interests of all road users. Traditional research, heavily reliant on discrete mathematical statistics methods like Logit/Probit, has yielded valuable insights into accident influencing factors [1]. Notably, Sheikh Manirul Islam et al. [2] used a hierarchical multinomial logit model to evaluate factors affecting severity of right-turn crash injuries at 221 signalized intersections, considering within-intersection crash correlation. Shen Xin et al. [3] employed the Multinomial Logit model to explore the severity of traffic accidents, highlighting significant contributing variables. Furthermore, Wang Peng et al. [4] employed the ordered Probit method to delve into the intricate causal relationships between a wide array of factors—encompassing environmental conditions, road attributes, vehicle specifications, and driver behavior—and the severity of rear-end collisions. Despite the proven effectiveness of discrete choice models in accident analysis, their utilization often neglects the inherent ordinality of accident severity. Moreover, the reality of incomplete accident records, imprecise indicator variables, and potential false positives can introduce biases into parameter estimations, undermining the accuracy of results [5]. Consequently, there is a pressing need to transcend these limitations and adopt advanced analytical approaches capable of capturing the complexity and nuances of traffic accident causality. This endeavor will not only advance our understanding of accident severity but also pave the way for the development of more effective and targeted traffic safety interventions.

Because of its own characteristics, the traditional discrete model not only requires high data quality, such as data collation in the data preparation stage, but also has restrictions on the conditions of utilizing. In order to solve the problems above, some scholars try to utilize non-parametric methods to analyze the influence factors of accidents, such as classification regression model, Bayesian network model, neural network model and many other models. Studies have shown that non-parametric methods show better statistical fitting ability and generalization performance in dealing with accident analysis problems. For example, Sun Yixuan et al. [6] established a decision tree model to analyze the differences of factors influence the severity of road accidents. Li Qingquan et al. [7] utilized fuzzy support vector machine (FSVM) to successfully predict the road accident status. Shi Juan et al. [8] utilized Back Propagation neural network to analyze the unsafe factors suffered by workers, and established a prediction model of accident injury severity, which achieved good performance. Nonetheless, it is crucial to recognize the limitations inherent in existing research: The sample datasets employed may be limited in size and lack of comprehensive records pertaining to casualties, injury locations, and vehicle conditions, as noted in the reference article. Furthermore, the categorization of road accident severity into a finite number of classes could be refined to offer a more intricate perspective on accident outcomes.

Despite these limitations, non-parametric methods offer a promising avenue for advancing beyond traditional discrete models. They empower researchers to delve deeper into the intricate web of factors influencing road accident severity, thereby informing the formulation of more targeted and effective traffic safety strategies. This paper employs an enhanced Support Vector Machine (SVM) model to analyze urban ring road accident data and uncover the impact of latent influencing factors on accident severity. Through the construction of various SVM models, including those with linear kernel functions, inhomogeneous polynomial kernel functions, and Gaussian radial basis kernel functions, this study comprehensively explores the effects of 16 potential influencing factors spanning the four primary aspects of humans, vehicles, roads, and the environment on accident severity. This approach can overcome the limitations of traditional discrete choice models, enhancing the accuracy of accident severity predictions. Additionally, it provides a fresh perspective and tool for traffic accident analysis, facilitating a deeper understanding of accident mechanisms and influencing factors. Based on the precise prediction model proposed in this paper, traffic management departments can develop more targeted safety strategies, thereby effectively reducing the severity of ring road traffic accidents and protecting public life and property safety.

## 2. Data description and processing

### 2.1 Data description

The data utilized in this paper comprises 1250 data records of traffic road safety accidents that occurred over the past 3 years on the urban ring roads of a central city in northwest China. As detailed in Table 1, these records encapsulate essential information pertaining to the accidents’ characteristics, the parties involved and their casualties, vehicle type specifics, and additional supplementary data. To ensure the utmost precision in analyzing the interplay between accident severity and its influencing factors, a rigorous data cleansing process was undertaken. This involved discarding attributes characterized by high missing rates, such as those related to the transportation of hazardous materials or vehicle modifications, along with attributes displaying low correlation with accident severity, like vehicle registration numbers, colors, and investigator details. Recognizing the paramount importance of the closed-loop driving system—encompassing drivers, vehicles, roads, and the environment—in shaping accident severity, these components were designated as independent variables for subsequent modeling, with accident severity itself serving as the dependent variable.

**Table 1:** List of influencing factors.

Primary indicators	Secondary indicators	Proportion/%	Primary indicators	Secondary indicators	Proportion/%
Pavement condition X1	Grade one and two pavement	35.0	Separation of motor vehicles and non-motor vehicles X10	Yes*	35.6
	Grade three and four pavement	40.0		No	64.4
	Others pavement *	25.0	Week day X11	holiday	52.2
Violation X2	Motor vehicle violation *	10.5		Working days *	47.8
	Non-motor vehicle violation	45.0	Age X12	<30*	47.5
	Pedestrian violation	44.5		30~55	33.2
Accident period X3	Morning and evening rush hours	25.0		>55	19.3
	Early morning hours	55.0	Road condition X13	Dry road surface *	45.3
	Other time periods *	20.0		Wet and slippery pavement	23.7
Vehicle type X4	Car	78.0		Waterlogged pavement	19.5
	Truck	22.0		Others	11.5
Accident type X5	Rear-end collision *	63.0	Season X14	Spring *	25.2
	Side collision	25.0		Summer	16.5
	Head-on collision	12.0		Autumn	20.1
Road lighting X6	Alternating light and dark	12.5		Winter	38.2
	Lighting at night	65.3	Driving distance X15	≤40000km *	65.3
	No lighting at night *	22.2		>40000km	34.7
Accident lane X7	Fast Lane *	25.3	Gender X16	Male	55.2
	Slow lane	35.1		Female *	44.8
	Inlet	39.6	Accident severity Y	Only property damage accident	32.1
Risky driving X8	Yes	59.5		Injury accident	55.4
	No *	40.5		Fatal accident	12.5
Visibility X9	≤50m	16.5	--	--	--
	50~200m	25.2	--	--	--
	>200m*	58.3	--	--	--

Note: Marked with * are reference groups.

### 2.2 Data processing

Data imbalance caused by excessive or rare proportion value of each potential influencing factors of accident severity will influence the analysis effect of the established model, and combining values with similar meanings can compensate for the impact of data imbalance [9]. In order to prevent the proportion of each value in the balanced attribute from being too little, the lower limit of the proportion of attribute values is set to 10% in this paper [10]. For example, due to the improvement of the current road maintenance and management level, "snow and ice" rarely occur on the road. Considering that the proportion of "snow and ice" in the road condition is less than the set lower limit, as shown in Table 1, it is merged into "others".

## 3. Theoretical methods

### 3.1 Data inspection

Reviewing the current research status, most scholars rely on the traditional discrete choice model-Logit/Probit model to analyze the severity of traffic accidents, but there is deficient work on the verification of the conditions for the utilizing of the discrete choice model. However the traditional discrete choice model in application is with strict applicable conditions, such as the Multinomial Logit model is applicable to the preconditions that require the independence of independent selection, while the ordered Logit model is applicable to the conditions that require the parallel line assumption. In most studies, the severity of road accidents is divided into “only property damage accident”, “injury accident” and “fatal accident”. With the help of SPSS, the Small-Hsiao [11] method is utilized to test the independence hypothesis of independent selection, and the Hausman [12] method is utilized to test the parallel line hypothesis. Following Tables 2 and 3 for the test results.

**Table 2:** Small-Hsiao independence hypothesis test.

Dependent variable	χ^2^value	DF	P value	True or false
**2**	16.517	28	0.535	Accepted H0
**3**	38.278	28	0.015	Reject H0

**Table 3:** Hausman parallel line hypothesis test.

Independent variable	χ^2^value	DF	P value	True or false
All	136.053	28	0.001	Reject H0

It can be seen from Tables 2 and 3 that the test results do not meet the conditions of independence hypothesis test of independent selection and parallel line hypothesis test, and the traditional discrete selection model cannot be utilized to analyze the accident data, Therefore, the related model in the non-parametric method is considered to analyze the data.

### 3.2 SVM model

SVM model is a non-parametric solution to classification problems based on statistical learning and reasoning knowledge. For binary classification problems, try to find a hyperplane to separate the uncertain number sets, where the hyperplane equation can be expressed as follows:

W·X+b=0 (1)

In the hyperplane set composed of hyperplanes, there is an optimal separation hyperplane. The separation hyperplane has a unique determination of the edge vector. Therefore, under the condition that the data is nonlinear and separable, the SVM model will be transformed into solving the optimal problem:

$$\begin{array}{l} \text{min}\left\{\left.0.5{\text{W}}^{\text{T}}\text{W}+c\sum_{i=1}^{N}\xi_{i}x\right\}\right.\\ s.t.y_{\text{i}}(W^{T}\varphi (x_{i})+b)\geq 1-\xi_{i},i=1,2\ldots n \end{array}$$ (2)

In the formula, parameters C and ξ_i_ respectively represent the penalty factor of classification error and the relaxation variable of classification error. However, the value of C should meet the above constraints.

The Lagrange operator is further introduced to obtain the corresponding classification decision function:

$$f(x)=\text{sign}\left[\sum_{\forall i,\alpha_{i}≻0}y_{i}\alpha_{i}(x_{i}\times x)+b\right]$$ (3)

In the Eq (3),αi, i, x and b respectively represent Lagrangian operators, support vectors and real numbers, where real number b is defined as the basic hyperplane. Since the data is linearly indivisible, it is necessary to map the non-fractional data to the high-dimensional eigenvector space by mapping it. In this case, kernel function can be utilized for nonlinear transformation:

$$k(x_{\text{i}}\times x_{j})=\varphi (x_{\text{i}})\times \varphi (x_{j})$$ (4)

For multi-classification problems to be solved, SVM is based on the idea of structural risk minimization, and realizes the processing ability of linear to nonlinear classification problems by constructing the kernel function to realize the mapping of feature space, and finally determines the optimal hyperplane in the mapping feature space to maximize the classification interval by changing the nature of the kernel function. Commonly utilized kernel functions are linear kernel function k_*l*_, non-homogeneous polynomial kernel function k_*p*_ and Gaussian radial basis kernel function k_*r*_, as shown in formula (5):

$$k_{l}(x_{i}\times x_{j})=x_{i}^{T}\times x_{j}$$ (5a)

$$k_{p}(x_{i}\times x_{j})={\left[\gamma (x_{i}\times x_{j})+1\right]}^{p}$$ (5b)

$$k_{r}(x_{i}\times x_{j})=\text{exp}(-\gamma {\left\|x_{i}-x_{j}\right\|}^{2})$$ (5c)

Combining formula (2), (4) and (5), it can be known that when the three different kernel functions are utilized, the corresponding parameters of the kernel function should be determined.

The SVM model itself cannot be directly utilized for multi-classification problems, and the multi-classification problems need to be processed step by step. Generally, there are three ways to deal with multi-classification problems using this model:

1. Cortes, based on K subcategories, first selects one category sample to be treated as one category, and the remaining K-1 categories are treated as another category, and constructs K SVM models step by step, so this method is called "one-to-other".
2. Knerr [13], based on the K classification problem, determines two classification categories each time, and only needs to design a two-classification SVM model for each two categories each time. This method is called "one-to-one".
3. Platt [14], with the help of decision tree theory, combines binary decision tree with SVM model to build a new multi-class recognizer.

## 4. Model verification and result analysis

### 4.1 Model verification

When utilizing SVM model to deal with multi-classification problems, the "one-to-one" method is widely utilized because of its own advantages. Therefore, the "one-to-one" method is utilized to analyze road accident data in this study. When the SVM model is tested repeatedly with three kernel functions, the average prediction accuracy of the classification model corresponding to the three kernel functions is the highest when the cross-validation adopts 10 fold cross. In addition to taking the average prediction accuracy of “only property damage accident”, “injury accident” and “fatal accident” in the severity of road accidents as the basis for judging the classification accuracy of the three kernel function SVM models, this paper also introduces F1 evaluation index based on the confusion matrix as the generalization performance evaluation standard of the model. As shown in Table 4, the prediction performance of SVM model with three kernel functions is obtained.

**Table 4:** SVM prediction performance corresponding to different kernel functions.

Kernel function type	Parameter	Training set performance/%		Test Set Performance/%	
		Accuracy	F1	Accuracy	F1
**Kl**	C = 7.0	57.5	75.2	58.6	76.5
**Kp**	C = 1.0, γ = 2.5, p = 0.6	71.3	78.1	73.1	80.3
**Kr**	C = 15.0, γ = 0.8	78.3	82.6	77.1	84.1

From the classification performance of the above three kernel functions, It is clear to know that the SVM model using Gaussian radial basis function has the highest classification accuracy. When dealing with nonlinear classification problems, the kernel parameters γand penalty factor C of Gaussian radial basis function influence the distribution of mapping space. The value of γ has a further impact on the number of support vectors. The more support vectors are obtained, the model is prone to under-fitting problems. However, the less support vectors are obtained, the model is prone to over-fitting problems [15]. These two parameters not only affect the training accuracy of the model, but also affect the training speed of the model. Therefore, the optimal kernel parameters γ and penalty factor C is extremely important [16].

### 4.2 Model parameter tuning

In order to further explore the impact of kernel parameters of Gaussian radial basis function on the classification performance of SVM models, three classification prediction models, BO-SVM, GS-SVM and RS-SVM, with Bayesian Optimization (BO), Grid Search (GS) and Rough Set (RS) as optimizers, are established respectively. The optimization parameters and prediction performance of the three models are shown in Table 5.

**Table 5:** Three improved SVM model performance.

Model name	C	γ	Training Set Performance (%)		Test Set Performance (%)	
			Accuracy	F1	Accuracy	F1
BO-SVM	19.5	0.25	85.1	89.9	87.5	88.6
GS-SVM	21.6	0.55	82.5	87.8	86.1	85.2
RS-SVM	24.3	1.53	79.8	81.5	80.6	84.2
Bilayer-LSTM	-	-	78.7	79.2	80.6	81.3
BP	-	-	79.2	81.3	81.3	82.1

By comparing Tables 4 and 5, it can be concluded that the performance of the SVM model based on the Gaussian radial basis function of the optimizer has been further improved. Comparing the classification performance of the SVM model obtained by the three optimizers, it can be seen that the performance of the BO-SVM model is the best compared with GS-SVM and RS-SVM. The conclusions can be drawn from the analysis of Table 5, the performance of the above three classification prediction models on the data set is good and the accuracy of the test set is more than 80%, but the performance of BO-SVM on the training set and test set is the best. In addition, the optimal parameters of the three models are obtained through 10 fold cross validation. Through the analysis of the optimal parameters, it can be seen that the parameters of the SVM model optimized by GS and RS are greater than that of BO, and the model is over-fitted, which ultimately leads to poor performance in the test set. The specific performance is that the effect of RS algorithm in optimizing parameters depends on the number of samples in the sample feature space. The sampling result is easy to make local optimization, and the optimization efficiency is low. However, GS algorithm cannot traverse all C and γ, in order to get the optimal parameter combination, we need to increase the grid search density and more computing resources.

To further explore the impact of kernel parameters in the Gaussian radial basis function on the classification performance of the SVM model, we established three classification prediction models: BO-SVM, GS-SVM, and RS-SVM, utilizing Bayesian Optimization (BO), Grid Search (GS), and Rough Set (RS) as optimizer, respectively. To comprehensively evaluate the performance of these models, in addition to comparing these three SVM variants, We also introduced two deep learning models of Double layer long short memory neural network (Bilayer-LSTM) [17] and Back-propagation Neural Network (BP), as comparative algorithms. All five prediction algorithms were trained on a Windows 10 system using Matlab R2023a. For robust model evaluation, this paper systematically split the HighD dataset into 8:1:1 for training, validation, and testing, and all models adopted 10-fold cross-validation. The deep learning environment was configured as follows: Deep Learning Toolbox 23.2 was used as the learning framework, running on hardware equipped with an Intel Xeon W-2295 CPU and an NVIDIA RTX A2000 GPU. For the deep learning models, we employed the Adam algorithm as the optimizer, with a learning rate set to 0.001, a dropout rate of 0.2 to prevent over-fitting, a batch size of 128, and 300 training epochs. Comparing Tables 4 and 5, we can draw the following conclusions.

1. The performance of SVM models with Gaussian radial basis kernel functions, aided by optimizer, has been further improved. Among the SVM variants, the BO-SVM model exhibits optimal performance compared to GS-SVM and RS-SVM. All five classification prediction models perform well on the dataset, with test set accuracy exceeding 80%. However, BO-SVM demonstrates the best performance in all aspects.
2. Through 10-fold cross-validation, the optimal parameters for the three traditional machine learning models, BO-SVM, GS-SVM, and RS-SVM were obtained. Analysis of these optimal parameters reveals that the SVM model parameters obtained using GS and RS optimization are relatively large compared to those obtained using BO. This results in model over-fitting, ultimately leading to poor performance on the test set. Specifically, the effectiveness of the RS algorithm in parameter optimization depends on the sampling frequency of the sample feature space, and the sampling results can easily lead to local optima with lower optimization efficiency. On the other hand, the GS algorithm cannot iterate through all possible combinations of C and γ [18, 19]. To obtain the optimal parameter combination, it requires increasing the grid search density, demanding more computational resources. These factors could explain why GS-SVM and RS-SVM do not exhibit optimal performance on the dataset.
3. Although the Bilayer-LSTM and BP models possess strong learning capabilities, their performance in this experiment did not surpass that of the BO-SVM model with parameter tuning. This can be attributed to several reasons: Firstly, considering the data scale and characteristics, when dealing with smaller datasets or less complex feature spaces, the potential of deep learning models may not be fully realized, whereas traditional machine learning algorithms like SVM perform more robustly and effectively in such scenarios. Secondly, due to their higher complexity, deep learning models have increased demands for data and computational resources. Limitations in resources or data may lead to overfitting or inadequate training, thereby affecting performance. Lastly, BO-SVM fine-tunes its kernel parameters through Bayesian optimization, giving it an advantage in specific tasks. In contrast, the performance of deep learning models relies more on the selection of hyperparameters and optimization strategies. And the performance of the BP model is slightly better than that of Bilayer LSTM because the dataset does not have strict time sequence data.

### 4.3 Analysis of potential influencing factors

#### 4.3.1 Sensitivity analysis of influencing factors

Explaining the influence of variables on response variables, commonly utilized methods include sensitivity analysis [20, 21], marginal influence coefficient [22] and backward selection method [23]. The principle of sensitivity analysis and marginal influence coefficient method is similar. Firstly, change the magnitude of each independent variable one by one while keeping the remaining independent variables unchanged. Secondly, the change of the average probability of different accident severity after each modification of the magnitude of the independent variable is calculated, to measure the relationship between potential accident impact variables and accident severity. The results of sensitivity analysis are shown in Table 6. However, the sensitivity analysis or marginal impact coefficient only reflects the single factor relationship between a certain potential impact variable and the accident severity, but cannot reflect the interaction of multiple impact variables on the accident severity. Therefore, sensitivity analysis is utilized to quantify the influence of a single influencing factor on the accident severity, and backward selection method is adopted to explore the influence of interaction between various influencing variables on the accident severity, and reveal the most influential variable combination on the severity of road accidents.

**Table 6:** Sensitivity analysis results.

Variable explanatory	Only property damage accident	Injury accident	Fatal accident	Variable explanatory	Only property damage accident	Injury accident	Fatal accident
Pavement condition X1				Visibility X9			
Grade one and two pavement	-0.0075	0.0101	0.0122	≤50m	0.0178	0.0097	-0.0532
Grade three or four pavement	0.0165	0.0063	-0.0128	50~200m	0.0086	0.0136	-0.0172
Violation X2				Separation of motor vehicles and non-motor vehicles X10			
Non-motor vehicle violation	0.0283	0.0095	-0.0053	No	0.0561	0.0432	-0.0063
Pedestrian violation	-0.0113	0.0305	0.0156	Week day X11			
Accident period X3				Holidays	-0.0065	0.0553	-0.0415
Morning and evening rush hours	0.0489	0.0233	-0.0605	Age X12			
Early morning hours	-0.0655	0.0125	0.0021	30~55	-0.0112	0.0073	-0.0063
Vehicle type X4				>55	0.0101	0.1503	-0.0293
Truck	-0.0289	0.0155	0.0263	Road condition X13			
Accident type X5				Wet and slippery road	0.0356	0.0278	-0.1032
Side collision	0.0205	-0.0017	-0.0101	Waterlogged pavement	0.0516	0.0038	-0.0542
Head-on collision	-0.0503	0.0715	0.0010	Others	0.1462	0.0895	-0.1278
Road lighting X6				Season X14			
Alternating light and dark	-0.0352	0.0532	0.0143	Summer	0.0243	0.0335	-0.0655
Lighting at night	0.0143	-0.0278	-0.0103	Autumn	0.0275	0.0423	-0.0565
Accident lane X7				Winter	0.0293	-0.0411	0.0035
Slow lane	0.0566	0.0432	-0.0232	Driving mileage X15			
Inlet	-0.0146	0.0412	0.0079	>40000km	0.0073	-0.0135	-0.0896
Risky driving X8				Gender X16			
Yes	-0.0108	0.0355	-0.0165	Male	0.0155	-0.0086	-0.0056

It is clear to know from Table 6 that the same potential influence factor has different significance effects on different accident severity degrees, and different potential influencing factors also have different significance influence on accidents of the same severity degrees [24]. The following is an analysis of the influence of potential factors on the severity degrees of road accident from four aspects: driver-vehicle-road-environment.

(1) Driving human factor. The age of drivers is divided into three stages, with the age group under 30 years old as the reference group. Compared with the reference group, the other two age control groups have reduced the accident death rate of 0.0063 and 0.0293 respectively, indicating that compared with young people, middle-aged people are less likely to cause serious accidents when driving, and middle-aged people are more cautious when driving; However, the proportion of injuries caused by middle-aged people compared with young people increased by 0.0073 and 0.1503 respectively, indicating that middle-aged people are more vulnerable to injuries than young people in accidents. Among the potential influencing factors of driver gender, taking female drivers as the reference group, it can be seen that the ratio of injury accidents and fatal accidents caused by male drivers has decreased by 0.0086 and 0.0056 compared with female drivers.

It shows that male drivers are more calm than female drivers in accidents and have better ability to deal with emergencies; However, the proportion of "only property damage accident" caused by male drivers is 0.0155 more than that of female drivers. This may be due to the fact that male drivers are more likely to drive “angry” vehicles when they encounter serious traffic jams during driving, such as forced congestion, forced lane change and other operations are more likely to cause "scratch" accidents. There is a deficiency in measuring whether a driver is a skilled driver by his driving age, it is more practical to use driving mileage as the measurement standard. Drivers with a driving mileage of 40000km can be considered as skilled drivers, and can avoid casualty accidents more than unskilled drivers. Taking unskilled drivers as the reference group, the ratio of casualty accidents caused by skilled drivers has decreased by 0.0135 and 0.0896 respectively, so the training intensity of drivers should be strengthened. Perfecting the training system of drivers and improving the cultivation of their own quality are very important for reducing the occurrence of injury and fatal accidents.

(2) Vehicle factor. Among the accidents caused by violation of rules and regulations, the ratio of property damage and injury accidents caused by violation of rules and regulations by non-motor vehicles increased by 0.0283 and 0.0095 respectively, taking motor vehicle violations (including driving without following the road markings, not giving way to pedestrians and running yellow lights, etc.) as the reference group, indicating that non-motor vehicles are more likely to cause only property damage accidents or injuries accidents to pedestrians or non-motor vehicle drivers. In addition, the rate of fatal accidents caused by non-motor vehicles decreased by 0.0053, indicating that non-motor vehicles are more likely to have accidents with non-motor vehicles or pedestrians; Compared with motor vehicles, pedestrian violations are more likely to cause serious injuries to pedestrians, indicating that pedestrians are the most vulnerable group of road users to serious injuries, and pedestrians should improve their safety awareness and self-cultivation. Among the accidents involving trucks, trucks are more likely to cause serious accidents and casualties. The ratio of casualties has increased by 0.0155 and 0.0263 respectively compared with cars. The reason is that trucks are more likely to cause serious injuries to the surrounding road users in the event of accidents due to their large inertia in driving. In order to reduce the consequences of serious accidents caused by trucks, the driving route and time of trucks can be reasonably limited to avoid driving during periods with large traffic volume. At the same time, the type of motor vehicle accident is also a potential influence factor of the severity of road accidents. Taking the type of rear-end collision accident as the reference group, the side collision is more likely to cause minor traffic accidents than the rear-end collision, which shows that the ratio of "only property damage accident" caused by rear-end collision has increased by 0.0205. However, compared with rear-end accidents, frontal collision accidents are more likely to cause casualties. Specifically, the ratio of casualties caused by frontal collision accidents has increased by 0.0715 and 0.0010 respectively. The reason for the increase in the probability of casualties in frontal collision accidents is that the driver or fellow passengers have weak traffic safety awareness and have not taken effective passive safety measures, such as the lack of safety belt wearing. Therefore, it is not only necessary to implement the safety belt wearing of the front seated passengers, but also particularly important for the rear passengers.

(3) Road factor. In addition, the road itself is also a potentially important factor that causes road accidents. Compared with the road without lighting conditions, the road with lighting conditions will reduce the rate of casualty accidents. The road with lighting conditions will provide the driver with a broader view at night. The driver does not need to turn on the high beam when driving on the road with good lighting conditions, which reduces the threat of dazzling, and even "instant blindness" caused by the high beam towards the driver. Compared with the impact of lighting conditions, the driver is also very sensitive to the light and dark changes. Especially in the urban tunnel section with saturated traffic flow, accidents in the tunnel are more likely to cause serious serial accidents of vehicles at the tunnel entrance. In order to prevent this phenomenon, the tunnel needs to consider the light change transition section at the tunnel entrance at the design stage to increase the driver’s adaptability to light changes. In addition, adding separation facilities between motor vehicles and non-motor vehicles in the same lane can reduce the ratio of injuries and fatalities to 0.0155 and 0.0265 respectively. Since non-motor vehicles are more vulnerable to injuries than motor vehicles when driving on the road, it is particularly important to isolate motor vehicles and non-motor vehicles on the same lane.

(4) Traffic environment factor. In addition, the traffic environment is also one of the potential factors that cause traffic accidents with different severity. The environmental factors listed in this study include weather factors, road surface factors, seasonal factors and holiday traffic flow factors. In the weather factors, taking the visibility greater than 200m as the reference group, by comparing the relationship between the severity of road accidents and the change of visibility, It is clear to know that with the reduction of visibility, the rate of casualty accidents has decreased to varying degrees. The reason is that with the reduction of visibility, drivers are more cautious in driving, and deliberately reduce the speed of driving, even if there is a traffic accident, the severity of road accidents will not be too serious. Changes in road conditions will also influence the driving speed of drivers. Taking dry roads as the reference group, slippery, waterlogged and even snowy and icy roads have drivers to reduce the speed compared with dry roads. Therefore, it is not difficult to understand that the latter three road condition will respectively reduce the ratio of fatal accidents by 0.1032, 0.0542 and 0.1278.

The saturation of traffic flow is also an important factor affecting the severity of road accidents. Due to the policy that vehicle license plates are not restricted on holidays in Chinese big cities, the traffic environment becomes more congested and the traffic flow becomes more saturated. However, due to lower vehicle speeds, the rate of fatalities and injuries decreases compared to workdays.

#### 4.3.2 Backward selection method analysis

The steps of the backward selection method are: Firstly, consider all potential influencing factors to obtain a 10 fold cross validation accuracy y_1_ for BO-SVM modeling. Secondly, rank the influencing factors according to their significance, as shown in Table 7, eliminate the influencing factors that are ranked lower in order according to the ranking order, continue to use BO-SVM modeling to obtain a 10 fold cross validation accuracy of y_2_ until all impression factors are eliminated and a series of prediction models M_i,j_ (i = 1,2,3; j = 1,2,…, 16) are obtained, Correspondingly, a series of accuracies y_i,j_ (i = 1,2,3; j = 1,2,…, 16) are obtained. The performance of the backward selection model is shown in Table 8.

**Table 7:** Influential factor significance ranking.

Only property damage accident		Injury accident		Fatal accident	
Influential variables	Ranking of significance	Influential variables	Ranking of significance	Influential variables	Ranking of significance
Accident type X5	1	Driving mileage X15	1	Age X12	1
Accident period X3	2	Risky driving X8	2	Accident type X5	2
Accident lane X7	3	Accident lane X7	3	Gender X16	3
Vehicle type X4	4	Gender X16	4	Vehicle type X4	4
Driving mileage X15	5	Vehicle type X4	5	Driving mileage X15	5
Week day X11	6	Age X12	6	Road lighting X6	6
Age X12	7	Pavement condition X1	7	Risky driving X8	7
Gender X16	8	Violation X2	8	Violation X2	8
Separation of motor vehicles and non-motor vehicles X10	9	Week day X11	9	Pavement condition X1	9
Violation X2	10	Separation of motor vehicles and non-motor vehicles X10	10	Accident period X3	10
Pavement condition X1	11	Season X14	11	Week day X11	11
Road condition X13	12	Road condition X13	12	Accident lane X7	12
Season X14	13	Accident period X3	13	Separation of motor vehicles and non-motor vehicles X10	13
Road lighting X6	14	Accident type X5	14	Season 14	14
Risky driving X8	15	Visibility X9	15	Road condition X13	15
Visibility X9	16	Road lighting X6	16	Visibility X9	16

**Table 8:** Backward selection method model performance comparison.

Only property damage accident			Injury accident			Fatal accident		
Model number	Model accuracy	P value	Model number	Model accuracy	*P value*	Model number	Model accuracy	P value
M_1,1_	88.79	—	M_2,1_	90.27	—	M_3,1_	87.53	—
M_1,2_	88.12	0.72	M_2,2_	89.76	0.78	M_3,2_	87.11	0.81
M_1,3_	87.56	0.65	M_2,3_	89.36	0.67	M_3,3_	86.31	0.72
M_1,4_	84.36	0.53	M_2,4_	88.49	0.60	M_3,4_	84.95	0.66
M_1,5_	80.95	0.35	M_2,5_	86.34	0.45	M_3,5_	81.33	0.53
M_1,6_	76.54	0.23	M_2,6_	83.26	0.26	M_3,6_	78.56	0.31
M_1,7_	70.32	0.11	M_2,7_	80.15	0.13	M_3,7_	75.65	0.13
M_1,8_	60.33	<0.05	M_2,8_	75.45	0.08	M_3,8_	71.26	0.06
M_1,9_	57.64	<0.05	M_2,9_	60.23	<0.05	M_3,9_	58.45	<0.05
M_1,10_	55.15	<0.05	M_2,10_	61.46	<0.05	M_3,10_	57.75	<0.05
M_1,11_	52.98	<0.05	M_2,11_	58.75	<0.05	M_3,11_	57.12	<0.05
M_1,12_	50.67	<0.05	M_2,12_	58.12	<0.05	M_3,12_	55.32	<0.05
M_1,13_	50.22	<0.05	M_2,13_	55.98	<0.05	M_3,13_	54.76	<0.05
M_1,14_	49.56	<0.05	M_2,14_	55.26	<0.05	M_3,14_	54.27	<0.05
M_1,15_	49.10	<0.05	M_2,15_	55.12	<0.05	M_3,15_	54.03	<0.05
M_1,16_	49.02	<0.05	M_2,16_	55.03	<0.05	M_3,16_	53.95	<0.05

Utilizing the backward selection method, the P value and accuracy rate of the "only property damage accident" model are generated. As shown in Table 8, there is a significant difference between the M_1,8_-M_1,15_ model and M_1,1_, and the accuracy rate of the model is reduced significantly. Therefore, the top seven core factors that affect the severity of “property loss accident” in Table 8 are: accident type, accident period, accident lane, vehicle type, driving mileage, week day, and age. Similarly, the top eight core factors affecting the severity of “Injury accident” are: driving mileage, risky driving, accident lane, gender, vehicle type, age, road conditions, and violations. The top eight core factors affecting the severity of “fatal accident” are: age, accident type, gender, vehicle type, road conditions, road lighting, risky driving, and violations.

Drivers are the largest uncertain factor influencing traffic operation safety. Some studies have found that human factors account for up to 90% of the factors related to traffic accidents [25, 26]. The influencing factors involved in this study include the age, driving mileage, and gender of the driver. The vehicle factors involved include the type of vehicle involved (trucks, cars, and non motor vehicles). Utilizing the backward selection method, age, driving mileage, and vehicle type were ultimately selected as the core influencing factors. It is clear to know that drivers’ physical functions also decrease to varying degrees as they age, such as their ability to obtain information, process information, and their agility. Measures such as driver health monitoring can be taken to protect the rights and interests of drivers, such as conducting regular physical examinations, and enforcing mandatory rest after driving for more than 4 hours continuously. With the increase in driving mileage, drivers’ ability to handle unexpected events and vehicle performance increases. It is recommended to increase the training time for truck drivers on the basis of the current driving test to further improve their driving skills and psychological quality. In addition, trucks account for a large proportion of the factors that cause serious casualties. It is recommended to limit the travel time and lane of trucks on conditional urban roads, and try to avoid large vehicle and pedestrian flows.

## 5. Conclusion

To analyze the potential influencing factors of the severity of traffic accidents on urban ring roads and provide theoretical support for the precise formulation of road accident improvement strategies, this paper explores the application of Support Vector Machine (SVM) models based on optimization strategies in predicting and classifying the severity of traffic accidents, mainly drawing the following research conclusions.

1. By employing a linear kernel function, a non-homogeneous polynomial kernel function, and a Gaussian radial basis function, we have validated the SVM model’s prediction and generalization abilities in addressing the multiple classification challenges associated with traffic accident severity. Notably, the SVM model, when equipped with a Gaussian radial basis function as its kernel, demonstrates superior performance. Furthermore, the introduction of the BO optimizer to enhance the BO-SVM model offers additional performance gains for the SVM approach.
2. The non-parametric BO-SVM model, rooted in Gaussian radial basis kernel functions, serves as a valuable adjunct to conventional discrete selection models, such as Logit/Probit. Through sensitivity analysis techniques, we’ve quantified the impact of 16 potential factors that encompass the integrated driver-vehicle-road-environment system on the likelihood of accidents of varying severity.
3. By adopting the backward selection technique, the paper precisely identifies the key factors that significantly influence the accidents severity levels for "only property damage accidents", "injury accidents," and "fatal accidents", this comprehensive analysis provides crucial insights into the determinants of accident severity, thereby enhancing our understanding of the factors that shape the outcomes of traffic incidents.

Although our study offers important contributions, we must also acknowledge its limitations. For example, the data samples selected in this study are insufficient, the sample data lacks detailed records related to casualties, and the injury site and severity are not explained in detail. The sample records lack detailed description of vehicle conditions. There are only three grades for the severity of road accidents, which is need to be further refined and improved.
